# Exploring the Role of Social Supermarkets in Addressing Food Insecurity and Food Waste: A Scoping Review

**DOI:** 10.1111/nbu.70053

**Published:** 2026-04-16

**Authors:** Claire O'Malley, Callum Bradford, Jordan Duffy, Helen J. Moore, Andrea Burrows, Joe Dunne, Lisa Harris, Frances Hillier‐Brown, Matthew Cotton, Lucie Nield, Amelia A. Lake

**Affiliations:** ^1^ School of Health and Life Sciences Teesside University Middlesbrough UK; ^2^ Fuse – The Centre for Translational Research in Public Health Newcastle UK; ^3^ School of Social Sciences, Humanities and Law Teesside University Middlesbrough UK; ^4^ Middlesbrough Environment City Middlesbrough UK; ^5^ Population Health Sciences Institute, Newcastle University Newcastle upon Tyne UK; ^6^ Advanced Wellbeing Research Centre, Sheffield Hallam University Sheffield UK

**Keywords:** food insecurity, health inequalities, social supermarkets, surplus food

## Abstract

Tackling food insecurity and food waste are two interrelated and complex policy challenges. Innovations in food retail termed ‘social supermarkets’ (SSMs) could provide a solution, utilising surplus produce from mainstream food retailers, which are then traded at reduced prices. This scoping review aimed to synthesise the peer‐reviewed research literature concerning the application of SSM models internationally, with a particular focus on how they influence food insecurity and reduce food waste. Free‐text keywords and index terms were combined with Boolean operators for comprehensive searches of MEDLINE, Embase, Cochrane, Web of Science, CINAHL, PsycINFO and Google Scholar. Fourteen studies were included after duplication removal, sifting and results from citation searches were added. SSMs were primarily used by low‐income, food‐insecure households, although some clients do not fall under these categories, instead choosing to shop at SSMs to prevent further societal food waste from unused surplus. Attitudes towards SSMs were predominantly positive (especially in comparison to food banks), largely due to the implementation of the choice model, where clients are treated as ‘customers’ rather than ‘recipients’ of food. Although SSMs play an important role in providing food assistance to the food insecure and preventing surplus food from supermarket retailers becoming food waste, they do not address the deeper underlying causes of food insecurity and food waste, namely: income inequality and the on‐going creation of surplus food throughout the food supply chain. Policies are needed which address these issues directly, unlike SSMs, which paradoxically rely on these increasingly dysfunctional systems to continue trading.

## 1 Introduction

The interrelated challenges of tackling food insecurity and reducing food waste remain two of the most complex societal challenges for public health researchers and policymakers to resolve. Food insecurity refers to a lack of consistent access to affordable and nutritionally adequate foods (Bickel et al. 2000). Prevalence of food insecurity is high among high‐income countries, with the United Kingdom, the United States and Australia reporting recent estimates of 8.0% (GOV.UK 2021), 2.8% (Rabbitt et al. 2023) and 13.4% (FAO 2019), respectively. The negative health and social impacts of food insecurity are numerous, with significant associations documented with asthma (Gundersen and Ziliak 2015), diabetes (Gucciardi et al. 2014), obesity (Morales and Berkowitz 2016), depression and stress (Pourmotabbed et al. 2020), poor sleep (Troxel et al. 2020) and adverse academic outcomes (Bruening et al. 2017). The origins of food insecurity are multi‐faceted, context‐specific and often the focus of broader political and civil society debate (Knezevic et al. 2014; Moraes et al. 2021; Pautz and Dempsey 2022). However, for more economically developed nations, evidence suggests that the two primary determinants of food insecurity relate to increasing economic inequality and neoliberal food policies that prioritise retail profit over issues of social security (Long et al. 2020).

Under conditions of financial vulnerability (brought about through successive financial and cost‐of‐living crises and government austerity measures), financially vulnerable groups increasingly rely upon emergency food aid provision to meet their nutritional needs (Strong 2020). Food banks, community kitchens and pantries are common models of community‐sector run emergency food aid provision that serve the needs of financially vulnerable people, including those with disabilities, people receiving both in‐work and out‐of‐work benefits and lone parents. Though food banks provide much‐needed assistance, they are often negatively perceived by users—with qualitative social research showing that individuals commonly feel embarrassed or ashamed to use them, citing their limitations in both the lack of choice and quality of foods provided (Purdam et al. 2016; Middleton et al. 2018). Moreover, emergency food aid organisations do not always equip users with the resources needed to resolve household food insecurity, as they lack the financial capacity to address its underlying causes (Middleton et al. 2018). Thus, from a food policy perspective, food banks are a double‐edged sword—they provide excellent assistance to vulnerable users while simultaneously entrenching voluntary and charitable food assistance as a core part of the “social safety net” of more economically developed nations (Loopstra 2018).

Running parallel to a crisis of food insecurity is the challenge of fully utilising food surplus, at both the production and retail stages of the supply chain to minimise waste. Food wastage continues to rise globally, with an estimated 670 million tonnes of edible food wasted across more economically developed nations each year (Amicarelli and Bux 2021). Food system challenges include; farm or fishery overproduction, inadequate food storage capacity, hospitality and household behaviours, social practices, supply chain disruption (including disruptions due to climate change) and product standards including stock control systems set by food retailers (e.g., not selling food near best‐before dates, damaged packing and misshaped fruit/vegetables) (Berri and Toma 2023; Saxena and Tornaghi 2018). A common policy solution to food insecurity is to create supply chain mechanisms which link food surplus to emergency food aid organisations, with the hope of simultaneously tackling both challenges. However, food given through charitable donation can stigmatise the recipient (Purdam et al. 2016). The mental health impact of food‐related stigma potentially outweighs the financial benefit for some vulnerable people (Bruckner et al. 2021; Middleton et al. 2018). Moreover, food relief met solely through supply chain surplus from retailers often produces a glut of seasonal produce, that may be culturally inappropriate or difficult to process in a domestic kitchen (e.g., large quantities of pumpkins in early November) or else is dominated by ultra‐processed, long shelf‐life food items with high quantities of sugar, saturated fats and salt that do not meet the nutritional needs of users (Webb et al. 2017). Resolving the interrelated challenges of managing food surplus types, retailer corporate social responsibility and the social practices of cooking, eating and producing waste within the home requires significant social innovation, moving beyond charitable food assistance, which reinforces cycles of social stigma and limited dietary choice.

### 1.1 Social Supermarkets (SSMs)

SSMs are an innovative and alternative food relief model to food banks. They differ in many ways, which are both complex and multifaceted. However, SSMs often promote that they tackle both food insecurity and food waste simultaneously (Papargyropoulou et al. 2022; Saxena and Tornaghi 2018); utilising surplus from mainstream food retailers to trade items in a conventional supermarket format but at greatly reduced prices (Berri and Toma 2023) (surplus in this context typically being food which is near a best before date, mislabelled, overstocked and/or has incorrect/damaged packaging). The unique combination of lower costs and a socially desirable customer experience (from a retail rather than charitable environment) has shown promise in meeting user needs for an affordable and dignified food procurement experience (Lindberg et al. 2019; Andriessen et al. 2022). Many SSMs tailor their services to the needs of local customers by offering voluntary/low‐cost training opportunities for various roles, in‐turn supporting clients to resolve household food insecurity (Booth et al. 2018) and/or providing a safe and inclusive venue to socialise, thus alleviating social isolation and loneliness commonly experienced by financially vulnerable people (McKay et al. 2018; Haines et al. 2018).

SSMs have great potential social value to the communities they serve. However, a systematic evaluation of their use in tackling food waste and food insecurity is lacking. Such a review is necessary to support evidence‐based food policy and practice in this new area of food assistance. This study, therefore, aims to synthesise the peer‐reviewed research literature concerning the application of the SSM model internationally and draw policy recommendations for food systems governance organisations and retail managers.

## 2 Methods

A scoping review of the peer‐reviewed research literature was conducted to understand the application and impact of the SSM model internationally. Reporting of the methodology and findings adheres to the Preferred Reporting Items for Systematic reviews and Meta‐Analyses extension for Scoping Reviews (PRISMA‐ScR) guidelines (Tricco et al. 2018). See Appendix SA for the completed checklist.

### 2.1 Eligibility Criteria

Studies were included if they incorporated primary data collection relevant to the phenomena of interest (SSMs) and were peer reviewed and published within an academic journal. No limits were applied to the sample, country, study design or publication year. Only studies published in English were considered.

For the purposes of this review, SSMs were defined as supermarkets which utilise surplus food (and potentially other goods) from more mainstream, larger retail stores to sell at a reduced price. SSMs should implement a choice model, allowing customers to choose and pay for their items as they would with a conventional supermarket. Studies exploring emergency food models which do not implement this choice model (e.g., food banks and emergency food parcels) were excluded. SSMs do, however, encompass a range of different names and models, including but not limited to: social enterprise models, food pantries, eco‐shops and community supermarkets.

### 2.2 Information Sources and Search Strategy

The search strategy was steered by the JBI guidance for scoping reviews (Peters et al. 2020). Free‐text keywords and index terms were combined with Boolean operators for comprehensive searches of MEDLINE (EBSCO), Embase (OVID), Cochrane, Web of Science, CINAHL (EBSCO), PsycINFO (EBSCO) and Google Scholar. Forward and backward citation searching was utilised following the first full‐text review of included studies. The search strategy for each database is outlined in Appendix SB. The search was conducted in October 2023.

### 2.3 Study Selection and Data Extraction

Authors C.B. (50.02%), A.B. (50.02%) and C.O. (100%) each independently screened a proportion of search results via titles and abstracts. Full text articles were later screened by C.B., A.B., C.O. and H.J.M. independently, with discrepancies resolved by consensus. Following the full‐text review, the identification and screening of studies via citation searching were conducted by C.O. and C.B.

### 2.4 Data Synthesis

The approach to synthesis was guided by the scoping review framework outlined by Arksey and O'Malley (2005). Key information was extracted from the included studies, charted thematically and presented in a narrative format.

## 3 Results

**FIGURE 1:**
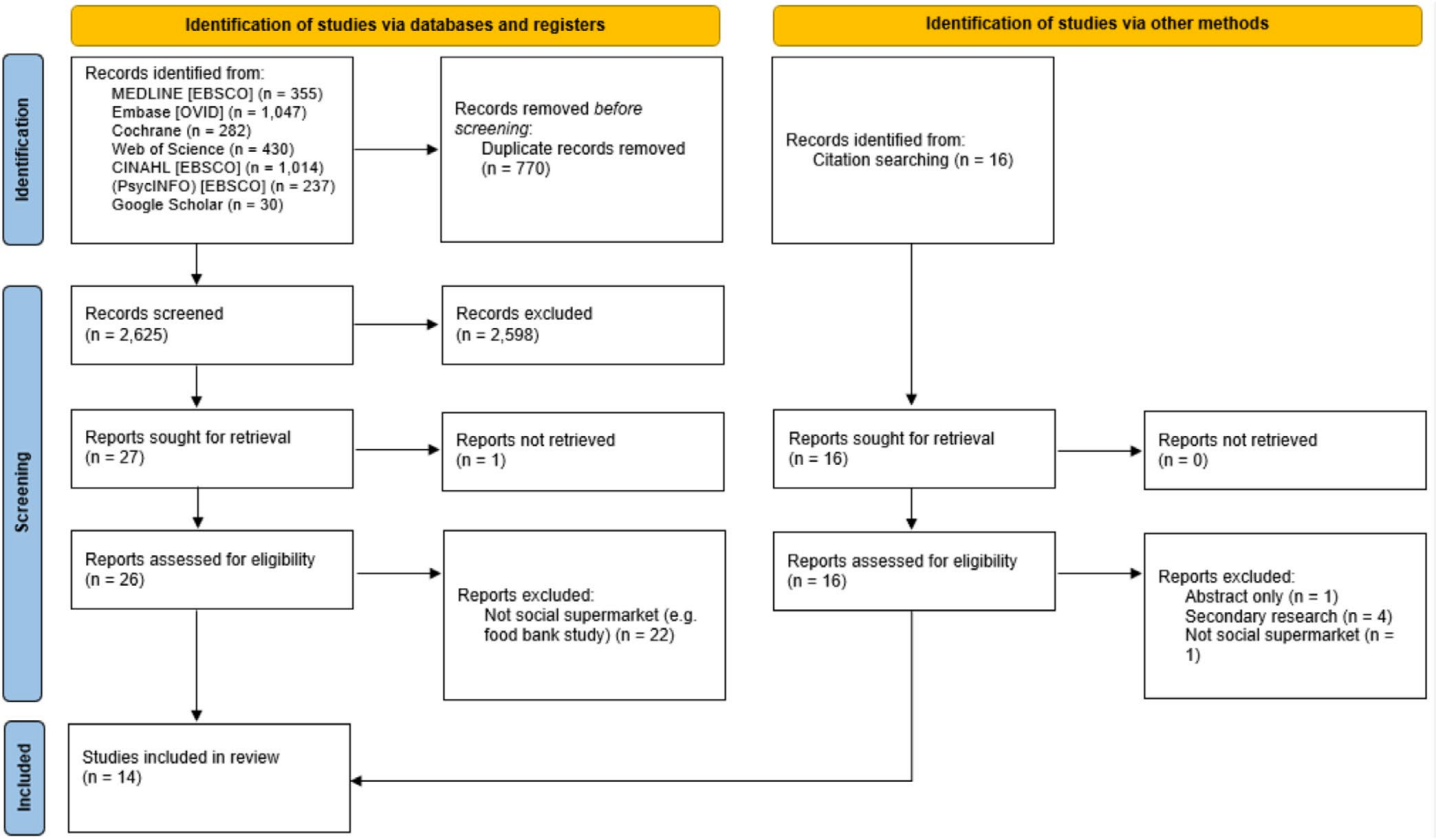
PRISMA flow diagram.

A total of 3395 records were identified from the initial database searches, with a total of 14 studies included after screening (which involved removing duplicates, sifting and adding studies found through citation searches; see Figure 1).

### 3.1 Characteristics of Included Studies

Across the 14 studies, research was conducted in a variety of countries, with some articles focusing on multiple locations. The most common location was Australia (*n* = 5) (Lindberg et al. 2019; Booth et al. 2018; Haines et al. 2018; McKay et al. 2018; Wills 2017), followed by the United Kingdom (*n* = 2) (Papargyropoulou et al. 2022; Berri and Toma 2023), Canada (*n* = 2) (Berri and Toma 2023; Lotoski et al. 2015), Austria (Lienbacher et al. 2021), Belgium (Andriessen et al. 2022) and Czechia (Sadílek 2021). The remaining two studies examined multiple countries, with one focusing on Eastern Europe (Croatia, Poland, Lithuania and Serbia) (Knežević et al. 2021) and the other spanning multiple continents (Europe, the United States, India and South Africa) (Michelini et al. 2018).

Studies employed a range of methods, most of which included the use of surveys and interviews with: SSM customers (Lindberg et al. 2019; Wills 2017; Haines et al. 2018; McKay et al. 2018), volunteers/employees of SSMs (Sadílek 2021; Wills 2017), stakeholders (Papargyropoulou et al. 2022) and the general public (Berri and Toma 2023; Knežević et al. 2021; Lotoski et al. 2015). Other methods included a hierarchical cluster analysis of SSM models (Michelini et al. 2018), focus groups with recipients of food relief services (Booth et al. 2018), ethnographic field work based at an SSM (Andriessen et al. 2022) and statistical analysis using data from SSM customer databases (Fuller et al. 2015). Study characteristics are outlined in Table 1.

**TABLE 1:** Characteristics of included studies.

Authors (year)	Primary research aim/question	Sample, data collection methods, and setting	Type of SSM reported (Y/N)	Definition/description of model stated
Lotoski et al. (2015)	To examine the awareness and use of an SSM 1 year after its opening	Door‐to‐door sampling of 365 residents in surrounding area of SSM	Y	The Good Food Junction: A non‐for‐profit full cooperative grocery store in a food desert
		Quantitative Survey		
		Canada		
Lindberg et al. (2019)	To examine an SSM's ability to increase access, use and availability of nutritious food in a socially acceptable way, for low socioeconomic status urban‐dwelling individuals	Comparative price audit (*n* = 27) at local stores (< 1 km), analysis of operational data from sample market (*n* = 3), customer surveys (*n* = 91) and customer interviews (*n* = 12)	Y	The Community Grocer: a social enterprise model offering a weekly pop‐up fresh food market
		Australia		
Fuller et al. (2015)	To examine healthy and less healthy food purchasing over a 1‐year period by shoppers, considering their proximity to an SSM	Analysis of 38 190 purchases and 583 SSM members' addresses	Y	The Good Food Junction: A non‐for‐profit full cooperative grocery store in a food desert
		Canada		
Booth et al. (2018)	To investigate the views of recipients of emergency and SSM food relief models	54 adult recipients of food relief	Y	Charity and social enterprise models
		Qualitative Focus Groups		
		Australia		
Lienbacher et al. (2021)	To illustrate the specific conditions of locational planning for SSMs in Austria (where SSMs are well‐established)	Authors collected a unique data set of 79 (2014) and 88 SSMs (2019), and 4665 (2014) and 4211 (2019) food retailers as (potential) suppliers to SSMs	Y	A social hybrid business model including private, public and non‐for‐profit
		Demand was determined using integrated wage and income tax data and unemployment rates (2011)		
		Austria		
Wills (2017)	To explore the absence of social enterprise responses to food insecurity in Australia	Semi‐structured interviews with key staff members from seven organisations involved in the Australian charitable food relief system	Y	Non‐for profit, social enterprise business model
		Quantitative survey with 38 consumers who accessed food via two charitable food pantries in Melbourne, Australia		
Andriessen et al. (2022)	To explore/understand how an SSM respects the dignity of food‐insecure people by approaching clients as customers in a grocery store setting	Ethnographic fieldwork was conducted over 7 weeks at SSM in Antwerp; 19 individuals were interviewed	Y	The social grocery model site is described as an SSM that has a welcoming room, utilises participatory methods, social activities and training
		Belgium		
Michelini et al. (2018)	Highlight how alternative distribution systems change in response to digitalisation and how the value propositions they claim change in the online context	Hierarchical cluster analysis based on sample of 52 food‐sharing cases	Y	The social grocery model (hybrid): Consisting of: Business to Consumer (B2C); Business to Business (B2B), Peer to Peer (P2P) and Consumer to Business (C2B)
		International (Europe, United States, India, South Africa)		
Knežević et al. (2021)	To measure attitudes towards the relevance and the role of SSMs in four central and eastern European countries	Web survey (*n* = 419)	Y	Small non‐for‐profit retailing operation
		Europe (Croatia, Poland, Lithuania, Serbia)		
Haines et al. (2018)	Explore the role of an SSM on the experience of food insecurity for people seeking asylum living in Melbourne, Australia	27 asylum seekers completed an interview (*n* = 12) or a survey (*n* = 15)	Y	Food for Justice Truck (FJT) social enterprise model
		Melbourne, Australia		
McKay et al. (2018)	Investigate the experiences of asylum seekers accessing an SSM	Twenty‐seven people seeking asylum completed a structured interview (*n* = 15) or a semi‐structured interview (*n* = 12)	Y	Food for Justice Truck (FJT) social enterprise model
		Melbourne, Australia		
Berri and Toma (2023)	To test and estimate relationships between factors that influence consumer intention to use an SSM	Quantitative Survey (*n* = 486)	N	N/A
		Representative sample of the UK population of shoppers of food products		
Papargyropoulou et al. (2022)	To critically examine the motivations, challenges and perspectives on surplus food redistribution (SFR) in the United Kingdom	*n* = 17 interviews; *n* = 40 2‐day workshop (UK SFR stakeholders)	N	N/A
Sadílek (2021)	To identify retail models redistributing suboptimal food (SSMs) and their presence in Czechia	40 SSMs observed (+5 interviews with store owners) in the Czech retail market in 2021	Y	Classified as SSM in accordance with the four main principles of Marketing, Behaviour, Voluntary and Benefit as described by Dann (2010)
		Store merchandise, prices, layout, employees and location noted		
		Czechia		

Four of the included studies utilised the term ‘social supermarket’ in describing the use of the SSM model (Berri and Toma 2023; Lienbacher et al. 2021; Knežević et al. 2021; Michelini et al. 2018). However, numerous other terms were also described, including: ‘social enterprise’ (noted in all five Australia‐based studies) (Wills 2017; Booth et al. 2018; Haines et al. 2018; McKay et al. 2018; Lindberg et al. 2019), ‘not‐for‐profit cooperative grocery store’ (Fuller et al. 2015; Lotoski et al. 2015), ‘surplus food redistribution’ (Papargyropoulou et al. 2022) and ‘social super discount stores’ (Sadílek 2021).

### 3.2 Awareness and Usage Data

In relation to overall awareness of SSMs, studies reported that most people were aware of their presence if one was available nearby. In Saskatoon, Canada, Lotoski et al. (2015) reported that, of 365 respondents living within the local vicinity of an SSM, only 5% (*n* = 20) were not aware of its existence. Of those that were aware, 69% (*n* = 251) had shopped there at least once (Lotoski et al. 2015). This aligns with later findings by Berri and Toma (2023), which suggested that those with greater knowledge of food assistance programmes exhibit stronger intentions to use them.

One study (Knežević et al. 2021) investigated the perceived purpose of the SSM model, finding heterogeneity between respondents and differences across countries. Respondents in Poland, for example, were more likely to associate SSMs with the reduction of food waste (65%, *n* = 80), while in Serbia, poverty reduction (49%, *n* = 53) was perceived as the main objective of the SSM model (Knežević et al. 2021).

Lindberg et al. (2019) investigated the employment status of customers shopping (*n* = 48) at an SSM, finding that approximately one‐third of customers (*n* = 17) were in full or part‐time employment. Additionally, Lotoski et al. (2015) reported that of those who had used an SSM at least once, 13% (*n* = 44) were unemployed, compared with 24% (*n* = 84) who were employed—the remaining identified with categories such as student, caregiver or retired. Haines et al. (2018) recorded a higher proportion of SSM users who were not employed and receiving social security benefits than those in employment; however, this was influenced by barriers to employment encountered by respondents resulting from their asylum‐seeking status (Haines et al. 2018).

Haines et al. (2018) also reported with their SSM of interest that 44% (*n* = 12) had experienced food insecurity at one point over the previous 30 days, half of whom had experienced food insecurity with hunger. Additionally, 55.5% (*n* = 15) of participants indicated instances of going to bed hungry, with one saying this happened ‘often’ (Haines et al. 2018). The SSM model documented by Haines et al. (2018) was created to support asylum seekers in Australia, as they were identified as being particularly vulnerable to food insecurity; however, 40% (*n* = 11) of participants reported eating less in Australia than they did in their home country (Haines et al. 2018).

Economically, the available evidence suggests that although most SSM users live in financially vulnerable households, a substantial minority do not. Lindberg et al. (2019) found that 20% (*n* = 10) of SSM users were not classified as living in a low‐income household. This suggests that users view SSMs not merely as emergency food suppliers (like traditional food banks), but as having a more positive atmosphere/environment, which the authors likened to a ‘farmer's market’. However, Lotoski et al. (2015) did identify a significant difference (*p* = 0.045) between the income of SSM shoppers and non‐SSM shoppers, with most of those visiting the SSM reporting an annual household income of less than 20 000 Canadian dollars, compared to the average annual household income of 56 000 at this time (Statistics Canada 2017).

Two studies found that SSM customers continued to access other food relief programmes due to food insecurity and availability (Booth et al. 2018; Haines et al. 2018), with Booth et al. (2018) reporting that participants (*n* = 54) had accessed nine different food relief sites in an attempt to overcome restrictions implemented on the frequency of visits and the amount of food that can be purchased. Although Haines et al. (2018) suggested that the SSM in question was not providing enough viable food options, leading to customers relying on alternative food sites, some of which were less costly.

Although some SSMs allow access to the general public, others have requirements or eligibility criteria for potential customers. Andriessen et al. (2022) reported that access to the social grocery was contingent on individuals having ‘250 euros or less to spend on food, clothes and other essential goods, after deducting expenses from monthly income’. Although the SSM described by Haines et al. (2018) and McKay et al. (2018) was designed to support asylum seekers, the models described in Booth et al. (2018) were available to a wide range of participants who had experienced hardship, such as unemployment, homelessness and having limited access to insufficient welfare funds.

### 3.3 Accessibility

Numerous studies (*n* = 4) (Fuller et al. 2015; Lindberg et al. 2019; Sadílek 2021; Knežević et al. 2021) referred to the locations of SSMs and the distance users had to travel to access them. Fuller et al. (2015) found that the average distance from home to an SSM for participants was 0.6 km (compared with 0.5 km for the nearest standard grocery store) in Canada, with Lindberg et al. (2019) reporting that 74.7% of shoppers lived within 1 km of the SSM in Australia. Sadílek (2021), who observed SSMs in Czechia, reported that 60% of stores were located in city centres, which was convenient for shoppers and subsequently attracted more visitors. However, this level of accessibility varies within and across countries. For example, when questioned about the presence of SSMs, 89% of participants in Lithuania reported having an SSM in their town/city, compared with only 8% of respondents in Serbia (despite 86% reporting a need for this type of food assistance model) (Knežević et al. 2021). Having an SSM available locally was highlighted as beneficial, given that the nearest major supermarkets often require the use of public transport or private vehicles, limiting their accessibility (Lindberg et al. 2019).

In relation to how shoppers travel to their local SSMs, findings were mixed. Lotoski et al. (2015) reported that 75% of shoppers walked to their SSM, with the figure rising to 80% among those for whom the SSM was their primary grocery store. By contrast, Haines et al. (2018) reported that most respondents (69%) used a personal vehicle to access the SSM. The remaining customers who did not have access to a vehicle and instead used public transport expressed how this impacts their ability to purchase larger volumes of food items, as it becomes difficult to transport a full shop home.

### 3.4 Availability and Affordability

Multiple studies (*n* = 5) (Fuller et al. 2015; Andriessen et al. 2022; Haines et al. 2018; Lindberg et al. 2019; McKay et al. 2018) highlighted the greater availability of, and user preference for fresh over tinned or frozen alternatives in SSMs. This provision is important as SMMs offer the opportunity to purchase foods that would normally be out of the user's budget in conventional supermarkets. McKay et al. (2018) noted how participants expressed ‘how thankful they were that the [social supermarket] stocked watermelon and strawberries’ as these were deemed ‘luxury items that would not be purchased at full price’.

However, not all SSMs provided fresh produce. Sadílek (2021) reviewed 40 stores across 5 retailers (all following an SSM operating model) and observed that 100% of these stores offered ‘beverages, dairy, tins and packets, and sweets’ and 57% (*n* = 23) offered ‘bread and bakery’ products. Frozen food products, including ‘frozen vegetables, as well as frozen meat and poultry’, were offered in 95% (*n* = 38) of stores and fresh meat and poultry in 70% (*n* = 28). None of the stores offered fresh fruit and vegetables. This was attributed to difficulties in acquiring good‐quality fresh produce from suppliers as well as the ‘demanding and complicated hygiene standards with which the retailers do not comply’ (Sadílek 2021). Haines et al. (2018) also reported a lack of the ‘full range of foods that participants required, including a larger selection of fruits, vegetables and legumes’.

Some studies found that SSMs needed to ration certain fresh food and ‘luxury’ items due to their limited availability. McKay et al. (2018) reported that ‘in‐season and abundant fruits and vegetables were available for customers to self‐select’, but that certain products like watermelon and rice were subject to ‘predetermined quotas’ and as such were not on display and had to be requested by customers. Participants commented that there was a desire for a wider range and greater availability of foods, but understood the attempts of the SSM to meet the needs of customers and ensure food is shared fairly (McKay et al. 2018). Andriessen et al. (2022) outlined the limited availability in relation to opening hours—customers could shop just once per week on a prescheduled day. They linked this to ‘organisational issues, such as funding to purchase more refrigerators’ suggesting that SSMs may be able to offer fresh produce on a more consistent basis if supported with greater funding.

Another key factor in ensuring that SSMs are beneficial to those using them is product pricing. Sadílek (2021) reported that the 40 stores offered discounted rates up to 95%, which equated to items being 50%–70% cheaper than average market prices in retail stores. Lindberg et al. (2019) also found that the SSM they evaluated was 41% cheaper for a comparable food shop than a local retail outlet, identifying through interviews that this was an important and well‐valued aspect of the store with its customers (Lindberg et al. 2019).

### 3.5 Use of the Choice Model

One of the key features of SSMs, which differentiates it from other food assistance models, is user choice, which the available evidence suggests helps create an empowering shopping experience. In Interviews with SSM users by Andriessen et al. (2022), no users reported having felt judged for their food choices, Participants highlighted that the opportunity to choose which items they wanted to purchase (an option that is not typically provided for emergency food banks), reduced feelings of guilt for not accepting certain items, while increasing feelings of autonomy (Andriessen et al. 2022). Additionally, the act of paying gives those visiting the SSM a sense of dignity, unlike visitors to food banks, who often report feelings of shame and embarrassment. This suggests that making a payment may help reduce the negative emotions associated with receiving food assistance that is often reported to be missing when attending food banks, negating the ‘expectation to perform and embody the status of the “deserving” food aid receiver’ (Andriessen et al. 2022).

Findings from two studies (Booth et al. 2018; Knežević et al. 2021) demonstrated that perceptions of SSMs are increasingly positive, which may influence intentions to shop there. Berri and Toma (2023), using structural equation modelling, demonstrate that more positive attitudes towards SSMs are strongly associated with greater intention to use. The effect size (0.63) was the strongest of all reported total effects on intention to use.

Booth et al. (2018) reported that when asked to rank social enterprise models, four out of seven focus groups placed SSMs first against traditional models such as vouchers, seated meals and food parcels. Participants perceived that SSMs were a socially acceptable model of food procurement, which also included opportunities for fostering community connection. They described SSMs as providing a ‘dignified eligibility process’ that did not regard those using the service as ‘recipients’ (Booth et al. 2018). These positive perceptions are crucial in the ability of SSMs to support communities, with Berri and Toma (2023) noting how those who perceive a greater sense of customer normalcy in using SSMs are less likely to experience negative emotions associated with doing so.

Though the choice model and the transactional nature of the SSM can strengthen user enthusiasm, it was found to occasionally have a negative impact on customers (McKay et al. 2018; Andriessen et al. 2022). For example, the SSM described by McKay et al. (2018) chose not to display item prices so that it was easier to alter or give discounts when deemed appropriate. However, the lack of transparency and availability of information meant that some customers did not feel comfortable approaching staff to enquire about the cost of items. This made it difficult for many customers to shop within their means, with some shoppers only discovering the total cost of their shop when at the checkout—subsequently having to return items they had selected to stay within their budget, which again might reinforce feelings of disempowerment or social stigma (McKay et al. 2018; Andriessen et al. 2022).

The SSM studied by Andriessen et al. (2022) imposed a weekly spending limit of €20 for individuals and €30 for families of two to four people. They provided guidance on how much could be spent on particular food types, specifying that only 10% of the total budget could be spent on premium or branded items, referred to as ‘special products’. Although the reason for these restrictions is to ensure equitable access to the discounted products, the study highlights how even with the choice model, not all SSMs are able to provide full autonomy to customers (Andriessen et al. 2022).

### 3.6 Tailoring to the Needs of Customers

Andriessen et al. (2022) reported that the SSM would utilise customer surveys to tailor the available products and meet the needs of customers. For example, paper towels were not initially made available due to environmental concerns; however, after customers expressed a need for this item, it was later made available (Andriessen et al. 2022). The demand‐driven approach of this SSM is further demonstrated through the removal of certain products when they are not being purchased and only accepting surplus donations if they meet the needs of the customers (Andriessen et al. 2022). These findings highlighted how only offering appropriate food protects individuals experiencing food insecurity ‘from the stigma of “being a lesser citizen” associated with receiving surplus food at food banks’ (Andriessen et al. 2022).

Tailoring of services to meet customer needs was demonstrated in some SSM models through flexible store opening times. For example, Lindberg et al. (2019) noted that variations in opening days and hours were ‘set to best suit the community at each site’, with certain locations closing during school holidays while others closed only on national public holidays. Such responsiveness was intended to ensure that food was ‘reliably and consistently available to local communities’ (Lindberg et al. 2019). However, this level of customer‐centred adaptation was not universal across all SSMs. In contrast, Andriessen et al. (2022) described limited availability in their study context, where customers were only able to shop once per week on a prescheduled day. They attributed these restricted opening arrangements to organisational constraints, including limited funding and insufficient refrigeration capacity to store fresh produce, suggesting that more consistent provision may be possible with greater structural investment. Taken together, these findings indicate that the degree of customer‐centric tailoring varies considerably between SSMs. While some models demonstrate flexibility and responsiveness to local needs, others operate within tighter resource constraints that restrict accessibility. This variability raises important questions about equity of access across the broader SSM model, as communities may experience markedly different levels of service depending on local funding, infrastructure, and organisational capacity.

Conversely, it may be suggested that SSM inadvertently imposes their own ideas on what they perceive customers to need. Andriessen et al. (2022) draw on Schwartz's (1967) concept of ‘socialising the gift receiver’ to argue that SSMs implicitly define and prescribe clients' needs according to organisational assumptions rather than engaging with users' lived requirements. This dynamic can limit the extent to which SSM services accommodate individual circumstances, a concern echoed in research on food aid systems showing that food parcels are often nutritionally inconsistent and fail to account for cultural and health‐specific dietary needs.

For example, systematic evidence finds that food bank parcels are frequently inadequate in meeting nutritional requirements and tailored individual needs, including culturally appropriate foods and health‐related diet preferences (Oldroyd et al. 2022). Studies in the Netherlands reveal that typical food bank parcels do not align with national healthy diet guidelines, which may undermine recipients' broader nutritional well‐being (Neter et al. 2016). Research also indicates that traditional pre‐packed food parcels often fail to meet the cultural, religious and medical requirements of users (Rizvi et al. 2021).

Within a UK context, analyses show imbalances in parcel content, namely high carbohydrate and sugar provision alongside inadequate micronutrients, suggesting that typical parcel contents do not consistently support a healthy, balanced diet (Fallaize et al. 2020). Although clients still describe themselves as customers of these services, this appears to coexist with a constrained sense of agency. Customers are reliant on these services for food security and accept the provision offered, even when it fails to meet their actual needs (Andriessen et al. 2022).

## 4 Social Benefits

In addition to providing affordable food, SSMs place considerable emphasis on creating a positive and welcoming environment for those who access them. Field observations of an SSM by Lindberg et al. (2019) describe a lively, community‐oriented atmosphere characterised by conversations between customers, volunteers and staff, alongside social and cultural activities such as barbeques, visiting artists, community services and vendors selling traditional handicrafts and culturally relevant foods. Reflecting this environment, 91.2% of customers reported that attending the SSM increased their sense of connection to the community (Lindberg et al. 2019). Interviews further suggested that the market facilitated social interactions that customers would not otherwise have experienced, distinguishing SSMs from conventional retail settings (Lindberg et al. 2019).

Consistent with these findings, customers in other studies described SSMs as offering value beyond access to subsidised food, instead functioning as spaces where they felt more comfortable and socially included (Haines et al. 2018; McKay et al. 2018). The social environment of SSMs was identified as particularly significant, with the provision of a regular time and place to engage with staff, volunteers and other customers, fostering friendships and enabling interactions grounded in dignity and respect (Haines et al. 2018; McKay et al. 2018).

Beyond social engagement, some SSMs also provide opportunities for training and personal development. Focus groups conducted by Booth et al. (2018) highlighted how SSMs support customers ‘beyond food’ through learning and training initiatives intended to create pathways out of poverty. Participants described these opportunities as a ‘stepping stone’ that ‘builds hope that I can get out of this position and get a bit better in life’ (Booth et al. 2018), underscoring the potential of SSMs to contribute to longer‐term social, economic, and community empowerment.

### 4.1 SSMs Going Forward

Three of the studies reviewed (Michelini et al. 2018; Wills 2017; Papargyropoulou et al. 2022) considered the future of SSMs, how they may develop and potential future areas of research, policy and practice to consider. Michelini et al. (2018) investigated the increasing number of food distribution models based online to understand whether digital technology changes each model or adds value. They observed that the SSM model was not found online in the same way it exists in person, perhaps suggesting that SSMs in their current form are heavily reliant on physical infrastructure and resources such as staff and volunteers. However, there are new online models emerging, such as Fiksuruoka.fi, which is in operation in Finland, an online supermarket focusing on reducing food waste while ensuring the availability of a range of affordable goods. This model may prove fruitful, but as of yet, there appear to be no current online platforms operated by existing SSMs, and those that do exist do not follow a common model/structure (Michelini et al. 2018).

Wills (2017) reported that Australia, at the time of writing, had not seen the development of SSMs in the same way as other economically comparable countries, despite having similar levels of food insecurity. They attributed this slow growth to a failure of market, government and voluntary sector innovation, primarily due to ‘resistance from dominant commercial players’ who ‘fear devaluing their product range despite the fact that food‐insecure clients cannot afford to shop in their outlets’ (Wills 2017). They suggest further work is needed to understand the conditions under which these models can thrive, given that they do not necessarily arise as a direct result of the existence and political acknowledgement of food insecurity (Wills 2017).

The redistribution of surplus food to those experiencing food insecurity is frequently framed as a ‘win–win’ solution, simultaneously addressing affordability concerns and reducing food waste. However, research by Papargyropoulou et al. (2022) questions the extent to which such models meaningfully resolve either issue. Drawing on interviews with stakeholders involved in surplus food redistribution, the authors argue that these approaches can ‘paradoxically reinforce the same problems they attempt to solve’ (Papargyropoulou et al. 2022). In particular, they highlight the failure of surplus redistribution initiatives to address the structural drivers of household food insecurity and food waste, alongside misaligned stakeholder motivations and persistent logistical and legal barriers that complicate effective redistribution.

An illustrative example from the UK is Too Good To Go (TGTG), a digital platform through which customers can purchase surplus food at a discounted price that would otherwise be discarded. While customers do not pay a membership fee, the platform remains profitable through annual and per‐sale commission charges to participating food businesses. Although schemes such as TGTG are often promoted as innovative solutions to food waste, they primarily function as market‐based interventions that benefit retailers by offsetting disposal costs and recapturing value from surplus stock. Crucially, these models do not necessarily serve those most affected by food insecurity, as access depends on digital literacy, upfront payment, mobility, and the ability to collect food within limited timeframes.

Moreover, surplus food platforms such as TGTG do not place food choice or nutritional suitability at their core, offering consumers little control over the contents of purchases. As a result, while such schemes may reduce waste within retail systems, they do little to challenge existing inequalities in food access or to provide dignified, needs‐based support for those experiencing food insecurity. This reinforces Papargyropoulou et al.'s (2022) critique that surplus redistribution models, rather than transforming food systems, may ultimately sustain the very conditions that produce both excess and deprivation.

## 5 Discussion

This review synthesises the peer‐reviewed research literature concerning the application of the SSM model to tackle food insecurity and food surplus waste across a range of more economically developed nations, and community development.

The consideration of non‐vulnerable users within SSMs raises important questions regarding the implications of user models for both equity and sustainability. On one hand, attracting ecologically conscious shoppers with greater financial capacity may enhance the financial viability and long‐term resilience of SSMs by broadening their customer base, increasing revenue streams, and reducing reliance on external funding or donations. Such cross‐subsidisation has the potential to stabilise operations and support continued access for food‐insecure households. On the other hand, the inclusion of non‐vulnerable users may risk diluting resources intended for those most in need, particularly where the supply of surplus food is limited, or pricing structures do not sufficiently prioritise financially vulnerable customers.

This tension highlights a core challenge for the SSM model: balancing financial sustainability with its social mission. Without clear safeguards, such as tiered pricing, targeted access mechanisms, or explicit prioritisation of vulnerable households, SSMs may inadvertently reproduce inequities in food access, favouring those with greater agency and purchasing power. Further research is therefore needed to examine how different SSM governance and pricing models mediate this trade‐off and whether hybrid user approaches ultimately strengthen or undermine the capacity of SSMs to address food insecurity.

Balancing of these motivations is what differentiates SSMs from emergency food banks—and unlike the food banks, social attitudes towards SSMs were predominantly positive. This was due to SSMs preserving dignity with a recognisable retail layout and clients being referred to as ‘customers’ rather than “recipients” of food assistance. Allowing customers to choose which items they wanted to purchase as opposed to being handed a pre‐packaged parcel of food, was also fundamental to these positive reviews.

Although the choice‐based model is frequently positioned as a more dignified alternative to traditional food aid, its limitations warrant scrutiny. Reported experiences of discomfort, such as embarrassment when querying prices, unclear signage or returning items at checkout due to insufficient funds, are better understood as operational failures of poorly implemented SSMs rather than inherent flaws of the choice model itself. Fundamentally, concerns focus on the limited scope of the choice model itself. Customers' options are shaped by inconsistent surplus supply, limited availability of fresh fruit and vegetables, and a disproportionate reliance on shelf‐stable, lower‐nutritional‐value foods compared to mainstream retailers. This is further impacted by operational constraints and policy and procedures, creating issues around dignity and customer identity, as restricted fresh produce availability may make the shopping experience less comparable to mainstream supermarkets, potentially undermining perceptions of choice and normality. Consequently, while it is clear that SSMs offer a degree of agency relative to pre‐packaged food aid, the choices available remain bounded by surplus‐driven provision rather than nutritional adequacy or individual need, calling into question the extent to which current models can deliver meaningful consumer choice in practice.

Evidence presented would suggest that SSMs can provide a more dignified approach to food assistance compared to other models. However, unlike food banks, SSMs do not provide emergency food aid to meet acute nutrition needs, and thus both models play important roles in the community food assistance sector. This point is reiterated in that customers of SSMs are also utilising other food relief programmes to meet their needs, especially regarding refrigerated foods, toiletries and cleaning products.

While the availability of locally situated SSMs was primarily perceived as beneficial, the spatial centralisation of these stores may inadvertently generate new barriers for certain population groups (Janatabadi et al. 2024). Although SSMs are often positioned in town centres or highly visible commercial hubs to maximise reach, this may disproportionately advantage residents living within walking distance, while disadvantaging those in peripheral rural or suburban areas. This may include individuals without access to a private vehicle, older adults, people with disabilities, low‐income households, and those with caring responsibilities (Keyes et al. 2025). The distance to a centralised SSM may remain prohibitive, even if it is closer than a major supermarket. To avoid transport‐related food access inequities, considerations which have shown promise in underserved communities such as mobile food markets, subsidised travel/services and equitable spatial planning (Appelhans et al. 2024; Kim and Erickson 2025; Zhang and Wang 2023).

Despite the positive reception of SSMs, it is important to note that as with food banks, they do not address the underlying causes of food insecurity, namely, neoliberal food policies that emphasise producer and retailer autonomy, broadening income inequality within society (Long et al. 2020; Papargyropoulou et al. 2022). In 2022, the World Inequality Report estimated that in Europe, the lower 50% of earners receive only 19% of regional income, with this figure even lower at 13% in North America and Asia (Chancel et al. 2022). This inequality of income is not expected to decrease without substantial policy intervention through strengthening progressive income and wealth taxation, increasing in and out of work financial benefits to financially vulnerable households (including but not limited to, carers, people living with disability and/or chronic of life‐limiting illness and lone parents) (Chancel et al. 2022; Evans et al. 2005; Long et al. 2020; Piketty 2014); a position which is commonly denied within government systems that favour market choice to resolve price and cost‐of‐living challenges faced by citizens (Evans et al. 2005; Piketty 2014). This inequality, combined with the responsibility to support those in need shifting from the state and onto the community sector, is understood to cause food insecurity (Long et al. 2020). With these causes in mind, the term ‘food poverty’ is sometimes preferred over food insecurity, as having insufficient economic access to nutritional food provides a more explicit description of the situation (in more economically developed nations), as opposed to ‘insecurity’, which may imply that the food itself is not abundant (Long et al. 2020). We express concern, therefore, that an SSM model could simply provide a veneer of respectability and social desirability to community food assistance, which in turn creates a barrier to the urgent policy action needed to address the underlying food poverty which drives the need for their existence.

Unlike food banks, some SSMs do offer routes out of food insecurity through various training opportunities (and by extension, further employment) (McKay et al. 2018; Haines et al. 2018), and this is one advantage of the SSM model of note. However, the extent to which SSM‐led training opportunities alleviate experienced food insecurity is unknown. Given their small scale and piecemeal implementation across the urban and rural food retail landscape, the impact of these social innovations is severely constrained in tackling the ‘deep poverty’ experienced across the most economically marginalised communities. SSMs have a range of potential benefits for alleviating food waste from the retail environment, though these must be tempered by case‐by‐case examination of food surplus quality, quantity and supply chain considerations. Retail supermarkets commonly earmark food as surplus for stock control reasons—surplus foods are usually past (or very close to) a ‘best‐before’ or ‘use‐by’ date, have been mislabelled or have damaged packaging. The redistribution of this food to people via SSMs or else sending it for use as animal feed (if they are not fit for human consumption) is often described as the most desirable option—surplus reuse is ‘at the top of the waste hierarchy’ (Department for Environment, Food and Rural Affairs 2024). Surplus redistribution and use is generally ecologically preferable to alternatives further down the pyramid, where surplus food becomes food waste and is then either recycled, incinerated or sent to landfill. However, the deeper problem of food surplus concerns over‐production, supply chain management and retailer profitability, which cannot be fully alleviated by food surplus redistributors further down the chain, a problem discussed in detail by Papargyropoulou et al. (2022). It is important to note that the surplus food SSMs utilise from supermarkets is a very small proportion of food waste (2% in the United Kingdom) (WRAP 2020), demonstrating the limited scale of influence that SSMs have in improving the sustainability of the food retail system. To substantially reduce food waste, policy attention should focus on producer and retailer responsibility for surplus food throughout the food supply chain (Papargyropoulou et al. 2022). Such polices should aim to prevent the systemic overproduction of food by imposing the transparent disclosure of food surplus by stakeholders, while confronting the on‐going cultural and economic reliance on overproduction (Messner et al. 2021; Papargyropoulou et al. 2022). Though SSMs provide a means to reduce surplus within food supply chains, they must not be used as greenwashing for an unsustainable food system.

### 5.1 Strength and Limitations

This study is the first to synthesise literature concerning the application of the SSM model relating to food insecurity and food waste across more economically developed countries. The review methods utilised here are fully replicable and can therefore be updated in the future to understand how the food redistribution environment has changed.

One limitation of this study relates to the search strategy. Searching the literature concerning SSMs is particularly difficult given (1) there is no widely agreed upon definition of SSMs and (2) SSMs go by many different names, some of which, ‘food pantries’ are shared by both SSMs and food banks. Some food banks may also implement elements of the SSM model by utilising surplus food or providing a choice of items, therefore blurring the line between food banks and SSMs. For research purposes, agreeing upon a universal definition of SSMs would be valuable; however, such a definition may not be useful to SSMs themselves, as one primary advantage to the community sector is their freedom to tailor themselves to the needs of locals, in turn making each SSM unique and less generalisable between each other.

## 6 Conclusions

SSMs are primarily used by financially vulnerable, food‐insecure households, although not all clients fall into these categories; some choose to shop at SSMs to reduce societal food surplus waste. Attitudes towards SSMs are predominantly positive, particularly when compared to traditional food banks. This is largely due to the implementation of the choice model, which treats clients as ‘customers’ rather than ‘recipients’ of food. However, SSMs do not fully address the underlying causes of food insecurity or food waste, namely, increasing income inequality and the persistent overproduction of surplus food.

Despite these limitations, SSMs play an important role in providing food assistance and diverting surplus from supermarket retailers that might otherwise become waste. Yet, there is little evidence that SSMs can sustainably alleviate food insecurity in the long term, and the food they rescue is negligible relative to the overall surplus generated across the food supply chain. Consequently, broader policy interventions aimed at reducing income inequality and surplus generation at the producer and retailer levels are essential to address the structural inequities and environmental inefficiencies of the current food system.

Looking forward, several considerations highlighted by the research could strengthen both future SSM models and their broader impact. First, SSMs need to prioritise geographical equity and accessibility to ensure that the benefits of the model reach those most in need. Determining the likelihood of what works best in different locations will also ensure services are context and customer‐specific, which inevitably involves working alongside, not just for the benefit of communities. Second, transparent and well‐communicated policies regarding rationing, eligibility, and pricing are crucial for maintaining the dignity that the transactional choice model seeks to uphold. Finally, the development of online SSM platforms could be instrumental not only in scaling operations but also in ensuring continuity of access for customers, particularly those with mobility or time constraints. By addressing these operational and structural considerations, SSMs can enhance their social value while remaining mindful of their limitations within an unjust and unsustainable food system.

## Author Contributions

**Claire O'Malley:** conceptualisation, formal analysis, funding acquisition, investigation, methodology, project administration, resources, supervision, validation, visualisation, writing – original draft, writing – review and editing. **Callum Bradford:** conceptualisation, formal analysis, funding acquisition, investigation, project administration, validation, visualisation, writing – original draft, writing – review and editing. **Jordan Duffy:** formal analysis, funding acquisition, writing – review and editing. **Helen J. Moore:** conceptualisation, formal analysis, funding acquisition, investigation, methodology, visualisation, writing – review and editing. **Andrea Burrows:** formal analysis, investigation, project administration, supervision, validation, visualisation, writing – review and editing. **Joe Dunne:** visualisation, writing – review and editing. **Lisa Harris:** writing – review and editing. **Frances Hillier‐Brown:** funding acquisition, methodology, writing – review and editing. **Matthew Cotton:** funding acquisition, methodology, writing – review and editing. **Lucie Nield:** writing – review and editing. **Amelia A. Lake:** conceptualisation, funding acquisition, methodology, validation, writing – review and editing.

## Funding

This work was supported by the National Institute for Health and Care Research, Applied Research Collaboration, North East and North Cumbria, UK (Grant number: OFC 2021_91).

## Conflicts of Interest

The authors declare no conflicts of interest.

## Supporting information


**Appendix SA:** PRISMA‐ScR checklist.

**Appendix SB:** Search strategy.

## Data Availability

Relevant data are available from the corresponding author upon reasonable request.
