# Supplementary material for: Exploring the Role of Social Supermarkets in Addressing Food Insecurity and Food Waste: A Scoping Review
Source: Nutr Bull. 2026 Apr 16;51(2):200–12. doi: 10.1111/nbu.70053 (PMC13254696; doi:10.1111/nbu.70053)
Supplement: Supplementary file 2 — Appendix SB: Search strategy. [file NBU-51-200-s002.docx]

**(EBSCO)**

**MEDLINE, CINAHL, PsycINFO**


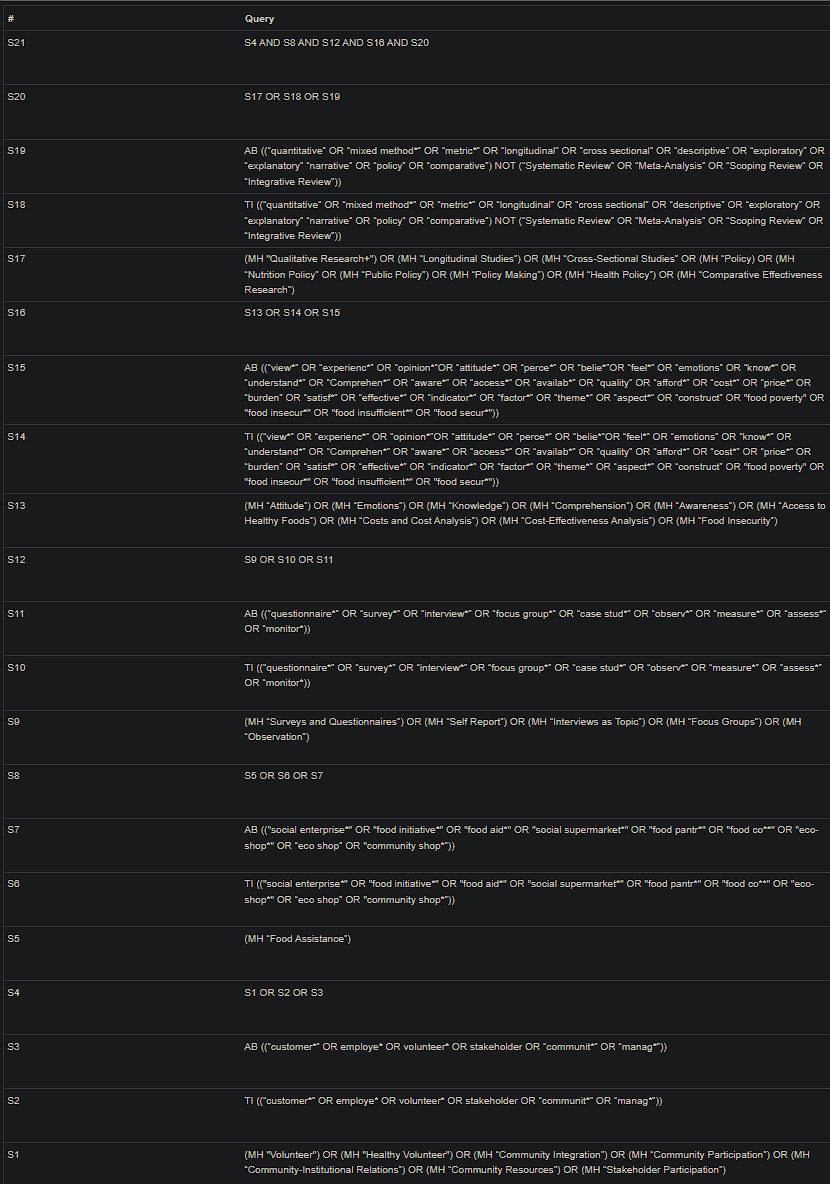


**(OVID)**

**Embase**

**
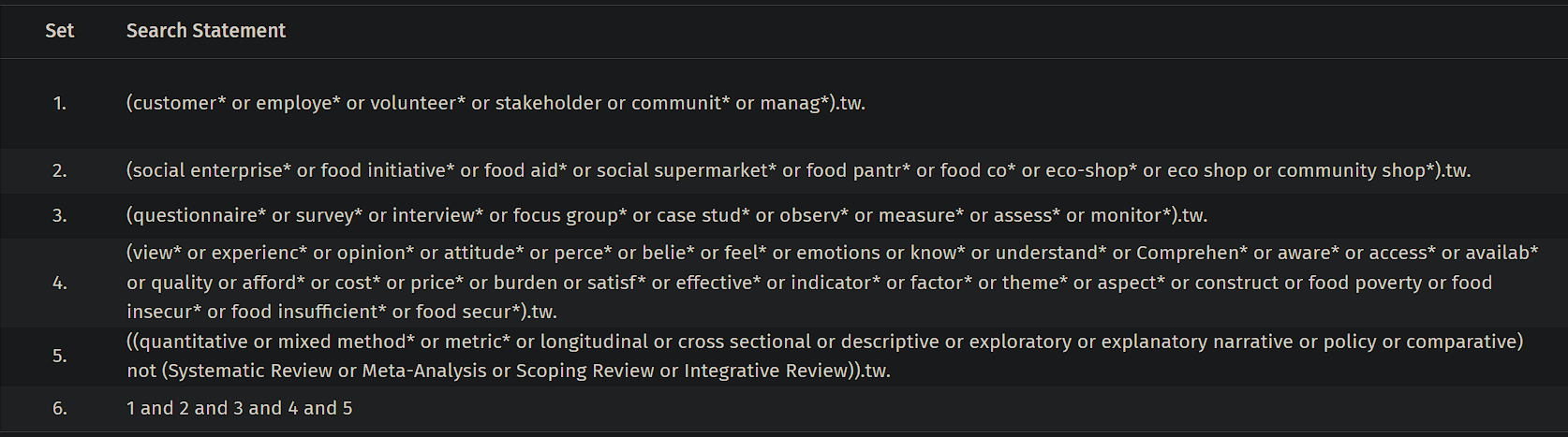
**

**Cochrane / Web of Science**

AB / TI (“customer*” OR “employe*” OR volunteer* OR stakeholder OR “communit*” OR “manag*”)

&

AB / TI ("social enterprise*" OR "food initiative*" OR "food aid*" OR "social supermarket*" OR "food pantr*" OR "food co*" OR "eco-shop*" OR “eco shop” OR "community shop*”)

&

AB / TI (“questionnaire*” OR “survey*” OR “interview*” OR “focus group*” OR “case stud*” OR “observ*” OR “measure*” OR “assess*” OR “monitor*”)

&

AB / TI (“view*” OR “experienc*” OR “opinion*”OR “attitude*” OR “perce*” OR “belie*”OR “feel*” OR “emotions” OR “know*” OR “understand*” OR “Comprehen*” OR “aware*” OR “access*” OR “availab*” OR “quality” OR “afford*” OR “cost*” OR “price*” OR “burden” OR “satisf*” OR “effective*” OR “indicator*” OR “factor*” OR “theme*” OR “aspect*” OR “construct” OR "food poverty" OR "food insecur*" OR "food insufficient*" OR "food secur*")

&

AB / TI (“quantitative” OR “mixed method*” OR “metric*” OR “longitudinal” OR “cross sectional” OR “descriptive” OR “exploratory” OR “explanatory” “narrative” OR “policy” OR “comparative”) NOT (“Systematic Review” OR “Meta-Analysis” OR “Scoping Review” OR “Integrative Review”)

**Google Scholar**

- The strategy for scholar was designed to identify any publications which may not have been picked up by the previous searches.
- Due to the limitations of scholar, a two simple searches were implemented, “social supermarket” and “social enterprise” (title only). The first 15 results from each search were saved.
